# Leptin From Fibro‐Adipogenic Progenitor Cells (FAPs) Regulates Masseter Muscle Disuse Atrophy and Ectopic Fat Accumulation

**DOI:** 10.1002/jcsm.70141

**Published:** 2025-11-26

**Authors:** Song Xinyi, Li Tingting, Liu Xiaoyu, Yan Hao, Li Qingchun, Liu Wei, Cheng Yuhe, Zhang Keke, Chen Yi, Gao Ang, Hong Yongfeng, Wang Yuanyin, Wu Tingting

**Affiliations:** ^1^ College & Hospital of Stomatology Anhui Medical University, Anhui Province Key Laboratory of Oral Diseases Research Hefei China; ^2^ Department of Stomatology Qingdao Special Servicemen Recuperation Center of PLA Navy Qingdao China; ^3^ Department of Rehabilitation Medicine, the Second Affiliated Hospital Anhui Medical University Hefei China

**Keywords:** disuse atrophy, ectopic fat accumulation, fibro‐adipogenic progenitor cells (FAPs), leptin, masseter muscle

## Abstract

**Background:**

Excessive fat accumulation in muscles with disuse atrophy may impair muscle physiologic function and exacerbate the progression of atrophy, with causes largely unexplored. Evidence indicates leptin plays a crucial role in regulating skeletal muscle fat metabolism. Masticatory muscle (a special skeletal muscle) atrophy often causes aesthetic and functional problems due to various occlusal factors. This study examines leptin's role and mechanism in masseter muscle disuse atrophy and explores leptin's source.

**Methods:**

A C57BL/6J mouse disuse atrophy model was established. The ameliorative role of leptin in masseter muscle lipid accumulation and atrophy and its local sources were explored by injection of exogenous leptin and nilotinib, which specifically induces leptin‐producing fibro‐adipogenic progenitor cells (FAPs). Transcriptomic sequencing revealed the molecular mechanism of lipid accumulation in the masseter muscle, which was validated by in vitro experiments.

**Results:**

Mice with masseter muscle disuse atrophy showed significant fat accumulation (triglycerides, TG: 3.750‐fold elevation, *p* < 0.001). Local leptin injection reduced masseter muscle fat accumulation (TG: 2.330‐fold reduction, *p* < 0.001) and atrophy index (MuRF‐1: 2.068‐fold reduction, *p* < 0.05). Transcriptomic analysis revealed downregulated PPAR lipid metabolism pathways, with significant repression of peroxisome proliferator‐activated receptor α (PPARα) and consequent phenotypic effects. Leptin significantly upregulated PPARα expression (mRNA: 10.814‐fold elevation, *p* < 0.001; protein: 1.843‐fold elevation, *p* < 0.001). PPARα silencing in C2C12 cells abrogated leptin's lipid‐lowering effect (TG: 3.903‐fold reduction abolished, *p* < 0.01). Fluorescence‐activated cell sorting (FACS)‐isolated FAPs expressed leptin mRNA. Nilotinib‐induced FAP apoptosis reduced local leptin expression (1.628‐fold reduction, *p* < 0.05) and exacerbated masseter muscle lipid accumulation and atrophy (MuRF‐1: 2.007‐fold elevation, *p* < 0.001).

**Conclusion:**

Disuse atrophy reduces leptin secreted by FAPs, triggering lipid accumulation that exacerbates muscle degeneration. This reveals the critical regulatory role of FAPs in maintaining masseter muscle homeostasis through leptin‐mediated mechanisms.

## 1 Introduction

Masseter muscle disuse atrophy is recognized as a degenerative muscle pathology caused by factors such as chewing‐side preference (CSP) [1, 2], missing teeth [3] or ageing and disease [4, 5]. This condition significantly affects masticatory function, temporomandibular joint health, facial morphology and quality of life [6, 7, 8, 9] and may have severe consequences for developing children [10, 11]. Research has shown that this atrophic process involves not only the direct degradation of muscle fibres but also complex metabolic reprogramming, particularly disorders of fat metabolism [12], which impair the normal contraction of skeletal muscle [13]. Typically, skeletal muscle contains only a small amount of fat; however, when free fatty acid (FFA) production is excessive and myocyte oxidative capacity is reduced, FFA is stored in non‐adipose tissues, resulting in ectopic fat deposits [14]. Fat accumulation in skeletal muscle varies across different pathological states. Fat accumulation occurs in two primary forms: within the skeletal muscle fibres and between the fibres [15]. The mode of ectopic fat accumulation in masseter muscle disuse atrophy remains unclear, and the underlying mechanisms require further investigation.

Leptin, a peptide hormone primarily derived from adipocytes, plays a well‐defined role in regulating energy homeostasis, including food intake and energy expenditure, through its action on the central nervous system [16]. In addition to its central actions, leptin receptors are widely expressed in peripheral tissues, contributing to the functional pleiotropy of this hormone [17]. Evidence suggests that leptin is involved in regulating fat metabolism in skeletal muscle. Leptin promotes FFA oxidation and reduces triglyceride (TG) storage in muscle tissue [18, 19, 20, 21]. Results also suggest that both ob/ob and fat‐free (FF) mice exhibit underdeveloped muscle mass and strength, with ob/ob mice being fat‐rich and FF mice being fat‐deficient [22]. Therefore, occlusal atrophy may not only result from the indirect effects of fat accumulation but also from an imbalance in leptin regulation, which may directly impact the maintenance of occlusal homeostasis. However, the direct biological relationship between leptin, fat accumulation and muscle atrophy in masseter muscle atrophy remains unclear. We hypothesized that the ectopic deposition of fat during the atrophy of the masseter muscle could be linked to the dysregulation of leptin signalling. Leptin plays a crucial regulatory role in governing lipid metabolism within the masseter muscle and preserving muscle homeostasis.

This study aimed to investigate the role and underlying mechanisms of leptin in regulating fat metabolism and muscle mass in the masseter muscle during occlusal atrophy development, as well as its local sources within the muscle. Unilateral molar extraction in mice was employed as an experimental model to simulate masseter muscle atrophy induced by chewing preference, to observe the role of leptin in regulating ectopic fat deposition and to identify its source in the masseter muscle. We expect to reveal the changing trend of leptin in the process of disuse atrophy, as well as its regulatory effect on the fat metabolism of the masseter muscle, to provide a new theoretical basis and therapeutic targets for the prevention and treatment of disuse atrophy of the masseter muscle.

## 2 Methods and Materials

### 2.1 Animal Models

Mice were housed in closed, pathogen‐free facilities and, after a 1‐week acclimatization period, were placed at a temperature of 22°C ± 2°C and a relative humidity of 50% ± 5% and fed and watered ad libitum under a 12‐h light–dark cycle. Male C57BL/6 mice (> 6‐weeks old) were used for all experiments. All mice were randomly grouped and anaesthetized with 1% sodium pentobarbital (50 mg/kg) for left unilateral molar extraction to induce left masseter muscle disuse atrophy. In contrast, control mice were anaesthetized without surgery. Local intramuscular injection of recombinant mouse leptin (1 μg/side/each, Novoprotein) or saline control was applied once daily for 4 weeks starting from the 4th week after unilateral molar extraction. Nilotinib administration was performed via intraperitoneal (IP) injection of 25 mg/kg of nilotinib solution (1.25 mg/mL of 10% DMSO/corn oil) in mice. Injections were given once a day for 7 days from the third week onwards. Negative control and experimental mice were given nilotinib treatment, and the blank and positive control groups were given the solvent (corn oil) only. At the end of each experiment, mice were deeply anaesthetized and then cervical dislocated and executed, and blood samples and masseter muscle tissue were collected. All animal use and euthanasia protocols were reviewed and approved by the Ethics Committee of Anhui Medical University.

### 2.2 Masseter Muscle TG Measurement

TG content in the masseter muscle was measured using the TG Content Assay Kit (Solarbio) according to the manufacturer's instructions. Isopropyl alcohol was added at a ratio of 100–200 μL per 10–20 mg of tissue. Homogenization was carried out using a tissue grinder under low‐temperature conditions centrifuged at 4°C for approximately 8000 g for 5 min, and the supernatant was taken for subsequent assays. The TG content in skeletal muscle was normalized to the corresponding muscle weight.

### 2.3 Western Blot (WB) Analysis

Whole‐cell extracts of masseter muscle were prepared using 1 mL of RIPA lysis buffer containing 10‐μg/mL phosphatase inhibitor, 10‐μg/mL protease inhibitor and 20‐μg/mL PMSF. They were ground with a tissue grinder to the point where no visible tissue mass was present and were lysed for 30 min on ice, followed by centrifugation for 15 min at 4°C/14000 rpm, and the supernatant was collected after centrifugation. Fifty micrograms of protein were loaded onto a 10% SDS‐PAGE gel and electrophoresed according to standard procedures. After transfer, the membrane was blocked with 5% skim milk powder in TBST (Millipore). The membrane was then stained with anti‐leptin (Abcam), anti‐MuRF‐1 (Santa Cruz Biotechnology), anti‐MAFbx (Santa Cruz Biotechnology), antimouse LEPR (CST), antimouse AMPK (Abcam), antimouse p‐AMPK (Abcam), antimouse CPT‐1a (R&D Systems) and antimouse GAPDH (CST) primary antibodies, which were incubated overnight at 4°C in Western primary antibody diluent (Beyotime). The blot was then incubated with a secondary antibody labelled with HRP. The signals were detected using a multifunctional imager (5200, Tennant, China). Grey value analysis was performed using image‐j software.

### 2.4 C2C12 In Vitro Culture and Transfection of siRNA PPARα (siPPARα)

C2C12 myoblasts were differentiated into myotubes in DMEM medium containing 2% horse serum and then used for subsequent experiments. Palmitic acid (PA, 250 μM) was used to treat the cells for 24 h to simulate the myocyte lipid accumulation model, while leptin (50 ng/mL) was also administered. The regulatory effect of leptin on PPARα was investigated by transfecting small interfering RNA (siRNA) targeting PPARα using Lipofectamine 2000.

### 2.5 Extraction of FAPs From Masseter Muscle by FACS

After mice were euthanized, the body surface was disinfected with 75% ethanol, and the masseter muscles of the hind limbs were isolated bilaterally aseptically and cut into pieces after exclusion of nonmuscle tissue. Tissue fragments were digested with 0.2% type II collagenase (1 g/4 mL) at 37°C for 1 h, and then passed through 100‐ and 40‐μm filters to obtain single‐cell suspensions sequentially. After supernatant removal by centrifugation at 500 g, erythrocyte lysates were treated for 3 min, followed by repeated washing with precooled washing buffer. Single‐cell suspensions were incubated with antibodies against PDGFRα (PE), CD31 (APC), CD45 (APC) and Sca‐1 (FITC) for 30 min on ice and protected from light, and finally, primary FAP cells were obtained by flow cytometry sorting and collected in 5‐mL flow tubes containing 1 mL of culture medium for mRNA extraction and subsequent culture.

### 2.6 ELISA

Mice were fasted for 12 h prior to sampling, anaesthetised, blood was collected and centrifuged at 3000 rpm for 10 min after blood coagulation to separate the serum for subsequent assays. Leptin ELISA Kit (R&D Systems) and Insulin (INS) ELISA Kit (R&D Systems) were used to detect the expression of leptin and Insulin in serum.

### 2.7 Histology and Immunofluorescence

Mice were sacrificed using the decapitation method. Excised masseter muscles were transferred to 20% sucrose/PBS for overnight incubation for subsequent OCT embedding and freezing. Frozen sections were prepared using standard methods. Antigenic heat repair was performed in citrate solution after 15‐min fixation with 4% PFA, followed by the removal of endogenous peroxidase with 3% hydrogen peroxide and blocking in PBS containing 10% normal goat serum for 1 h at room temperature. Tissues were stained overnight at 4°C using anti‐leptin (Abcam) and antimouse PDGFRα (Santa Cruz Biotechnology) primary antibodies diluted in PBS. Primary antibodies were detected using secondary antibodies conjugated to Alexa Fluor 488 and Alexa Fluor 594. Fluorescence microscopy was performed using a German Leica orthostatic smart microscope DM6 B equipped with 488‐, 568‐ and 633‐nm lasers.

### 2.8 Immunohistochemistry

Excised fresh masseter muscles were fixed in 4% PFA overnight. The following day, paraffin embedding was prepared using a German Leica Tissue Dehydrator ASP300 following a standard sequence of ethanol dehydration steps. The prepared paraffin sections were deparaffinized in standard steps and sequentially subjected to antigen repair, removal of endogenous peroxidase and sealing with 10% goat serum. Tissues were stained overnight at 4°C using anti‐MuRF‐1 (Santa Cruz Biotechnology) and anti‐MAFbx (Santa Cruz Biotechnology) primary antibodies diluted with PBS. The tissues were also incubated with a secondary antibody labelled with HRP, followed by DAB staining and finally dehydrated and sealed with haematoxylin staining. Photographs were taken with a German Leica orthostatic smart microscope DM6 B.

### 2.9 Tunel Staining and Tunel Co‐Staining With PDGFRα

Paraffin sections were applied, deparaffinized and stained using the one‐step TUNEL Apoptosis Detection Kit (green fluorescence) (Beyotime) according to the manufacturer's instructions. Sections requiring co‐staining with PDGFRα were stained with TUNEL after sealing with 10% goat serum, followed by immunofluorescence staining according to the standard procedure for primary and secondary antibodies. Fluorescence microscopy was performed using a German Leica orthostatic smart microscope DM6 B equipped with 488‐, 568‐ and 633‐nm lasers.

### 2.10 Oil Red O Staining

TG accumulation in frozen sections of masseter muscle was stained with a modified oil red O staining kit (Solarbio). Fat accumulation was photographed with a Leica orthostatic smart microscope DM6 B from Germany.

### 2.11 RNA Sequencing and Analysis

Transcriptomic Analysis of the Mouse Masseter Muscle by RNA Sequencing (RNA‐seq). Sequencing of mouse eukaryotic transcriptome was entrusted to Nanjing Paysono Gene Technology Co. The mouse masseter muscles were removed and stored in liquid nitrogen for analysis. After RNA extraction, purification and library construction, the libraries were subjected to paired‐end (PE) sequencing using next‐generation sequencing (NGS) based on the Illumina sequencing platform. The filtered reads were compared with the reference genome using HISAT2 (http://ccb.jhu.edu/software/hisat2/index.shtml), an upgraded version of TopHat2. HTSeq (v0.9.1) was used to statistically compare the read count value of each gene as the raw expression of the gene. Gene expression levels were normalized using FPKM to ensure comparability across genes and samples. Differential gene expression analysis was performed using DESeq, and the conditions for selecting differentially expressed genes were expression fold difference |log2FoldChange| > 1 and significance *p*‐value < 0.05. Enrichment analysis of KEGG pathways was carried out using the cluster profile (v4.6.0) software, focusing on the significantly enriched pathways with a *p*‐value < 0.05. Gene set enrichment analysis was performed using GSEA (v4.1.0) software. The data discussed in this article have been deposited in NCBI's SRA database and can be accessed via BioProject ID: PRJNA1193868.

### 2.12 Statistical Analysis

No efficacy calculations were performed to predetermine sample size. We did not use any specific randomization method to determine how samples and animals were assigned to experimental groups and processed. Analyses were not performed in a blinded manner. No samples, mice or data points were excluded from the reported analyses. All data are expressed as mean ± standard error (mean ± SEM). Statistical methods of Student's *t*‐test and one‐way ANOVA were used, and differences were considered statistically significant at *p* < 0.05. All statistical analyses were performed using Prism 9 for Windows 11 (GraphPad Software, La Jolla, CA, USA).

## 3 Results

### 3.1 Unilateral Molar Extraction Induces Progressive Masseter Muscle Atrophy and Ectopic Fat Accumulation in Mice

**FIGURE 1:**
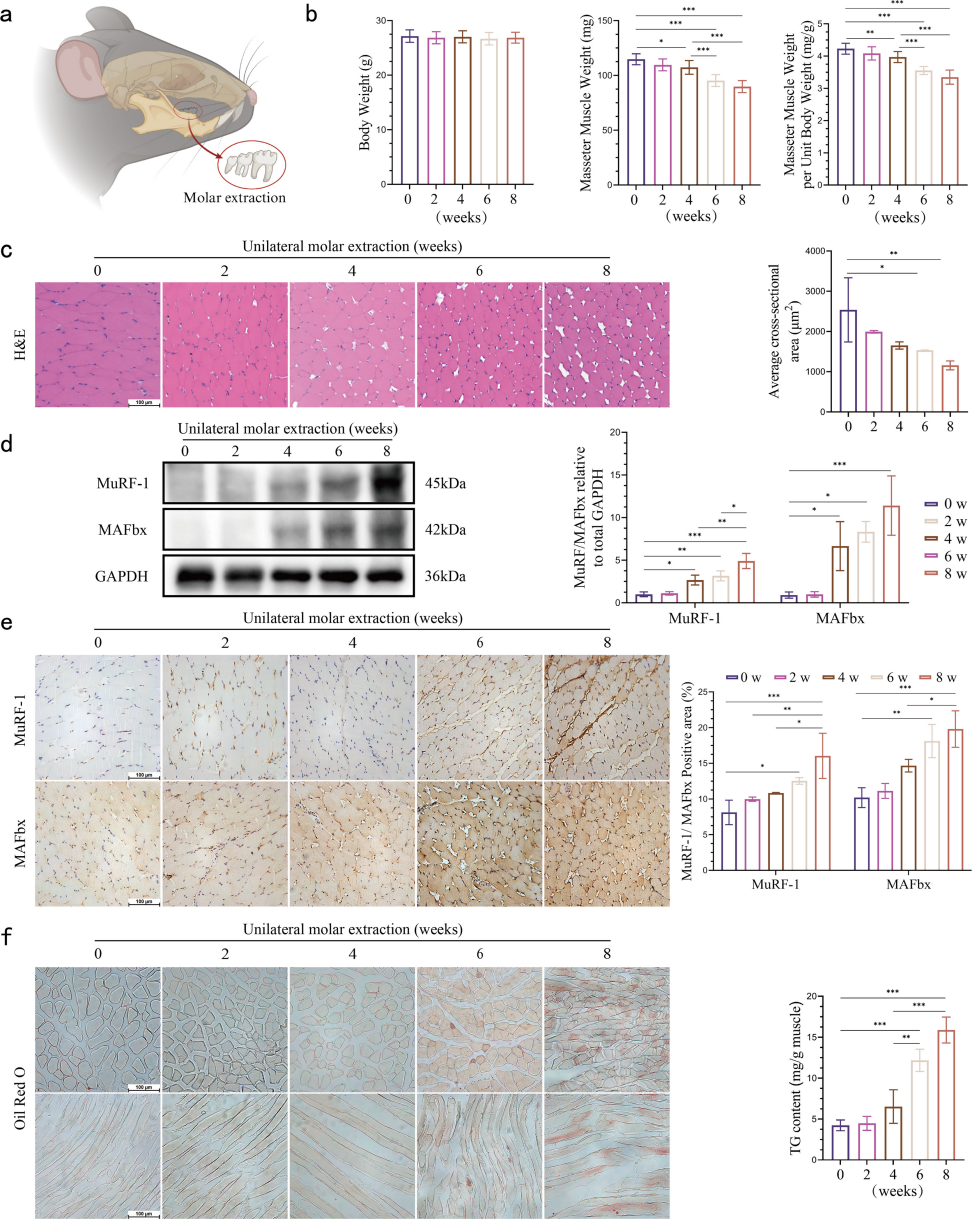
Masseter muscle atrophy and fat accumulation after unilateral molar extraction. (a) Schematic diagram of a unilateral extraction model. (b) Body weight of mice (g), mass of masseter muscle on the extraction side (mg) and mass per unit of body weight of masseter muscle on the extraction side (mg/g) (one‐way ANOVA; *n* = 12). (c) Quantitative analysis of H&E‐stained images and mean cross‐sectional area of muscle fibres of masseter muscle on the extraction side of mice (scale bar, 100 μm; one‐way ANOVA; *n* = 3). (d) MuRF‐1 and MAFbx bands and quantitative analysis in whole muscle extracts of masseter muscle on the extraction side of mice, GADPH was used as a loading control (one‐way ANOVA; *n* = 3). (e) Images of immunohistochemical staining (MuRF‐1 and MAFbx) of masseter muscle on the extracted side of mice and quantitative analysis of positive areas (brown) (scale bar, 100 μm; one‐way ANOVA; *n* = 3). (f) Image of Oil Red O staining of masseter muscle on the extracted side of mice (scale bar, 100 μm). (f) Triglyceride (TG) content of the masseter muscle on the extracted side of mice (one‐way ANOVA; *n* = 3). *p < 0.05, **p < 0.01, ***p < 0.001; error line, mean ± standard deviation. 0, 2, 4, 6 and 8 weeks: 0, 2, 4, 6 and 8 weeks after unilateral molar extraction.

The experimental mice underwent unilateral molar extraction to simulate the pathological process of disuse atrophy in the masticatory muscles (Figure 1a). The mass of the masseter muscle of mice on the molar extraction side was assessed at four different time points: There was no significant reduction in the weight of the masseter muscle at the beginning of the disuse period; the masseter muscle on the operated side showed a significant loss of mass due to the lack of practical occlusal function in the 4th week, and the loss of mass was more pronounced at 6 and 8 weeks. No significant difference in body weight was observed between the groups of mice during this period, as unilateral molar extraction did not affect regular feeding (Figure 1b). In addition, there were no significant differences in systemic metabolic indices among the groups of mice (Figure S2).

To assess the histologic changes in the masseter muscle on the molar extraction side, H&E staining was performed. With the passage of time, the masseter muscle fibres showed a progressive disorganization of the fibre arrangement and a significant enlargement of the muscle fibre gap after tooth extraction. The cross‐sectional area of the masseter muscle fibres at the 6th and 8th week after tooth extraction was significantly reduced compared with that before tooth extraction. This trend of reduction was progressively aggravated (Figure 1c). These findings were consistent with the typical histological features of skeletal muscle disuse atrophy.

To further assess whether the atrophy of the masseter muscle on the molar extraction side was related to muscle atrophy, western blotting was used to detect the expression levels of the muscle atrophy‐associated protein, such as MuRF1 and MAFbx. The protein expression of MuRF‐1 and MAFbx in masseter muscle increased significantly in the fourth week after tooth extraction and gradually increased until the eighth week. This suggests that muscle degradation is activated (Figure 1d). Immunohistochemical staining analysis also confirmed these findings. The expression of MuRF‐1 and MAFbx in the masseter muscle on the extraction side was significantly higher than that in the normal occlusal muscle at the eighth week after extraction (Figure 1e). Together, these data suggest that mice with unilateral molar extractions develop a habit of biased mastication (more use of the nonsurgical side for chewing), which leads to consumptive atrophy of the masseter muscle on the extraction side.

To assess ectopic fat accumulation during masseter muscle disuse atrophy, frozen sections were stained with Oil Red O. Almost no staining by Oil Red O was visible in the control group. In contrast, from the sixth week onwards, myocytes with Oil Red O staining started to enhance in the masseter muscle tissue. Severe fat infiltration was observed in half of the myocytes in the eighth week. However, the fat infiltration was confined to the intracellular region of the masseter muscle cells and did not extend into the interstitium nor result in the formation of new adipocytes (Figure 1f). The result of the content in skeletal muscle determined by quantification of TG exhibited a similar trend (Figure 1f). These findings indicate that mice with unilateral molar extractions undergo eccentric mastication, with progressive disuse atrophy of the masseter muscle on the side of the extracted tooth. This disuse atrophy leads to intracellular accumulation of fats in the muscle cells, which manifests in the later stages of the condition rather than at the beginning or during the early stages.

### 3.2 Leptin Deficiency as a Potential Cause of Ectopic Fat Accumulation and Muscle Atrophy in the Masseter Muscle

Leptin was the first adipokine identified to regulate fat metabolism and inhibit fat synthesis. The role of leptin in skeletal muscle is now progressively being studied more extensively, and there is a clear tendency for leptin to be upregulated during skeletal muscle regeneration [23]. We speculate that ectopic fat accumulation in masseter muscle atrophy may be related to abnormal regulation of leptin and further contribute to the development of muscle atrophy.

**FIGURE 2:**
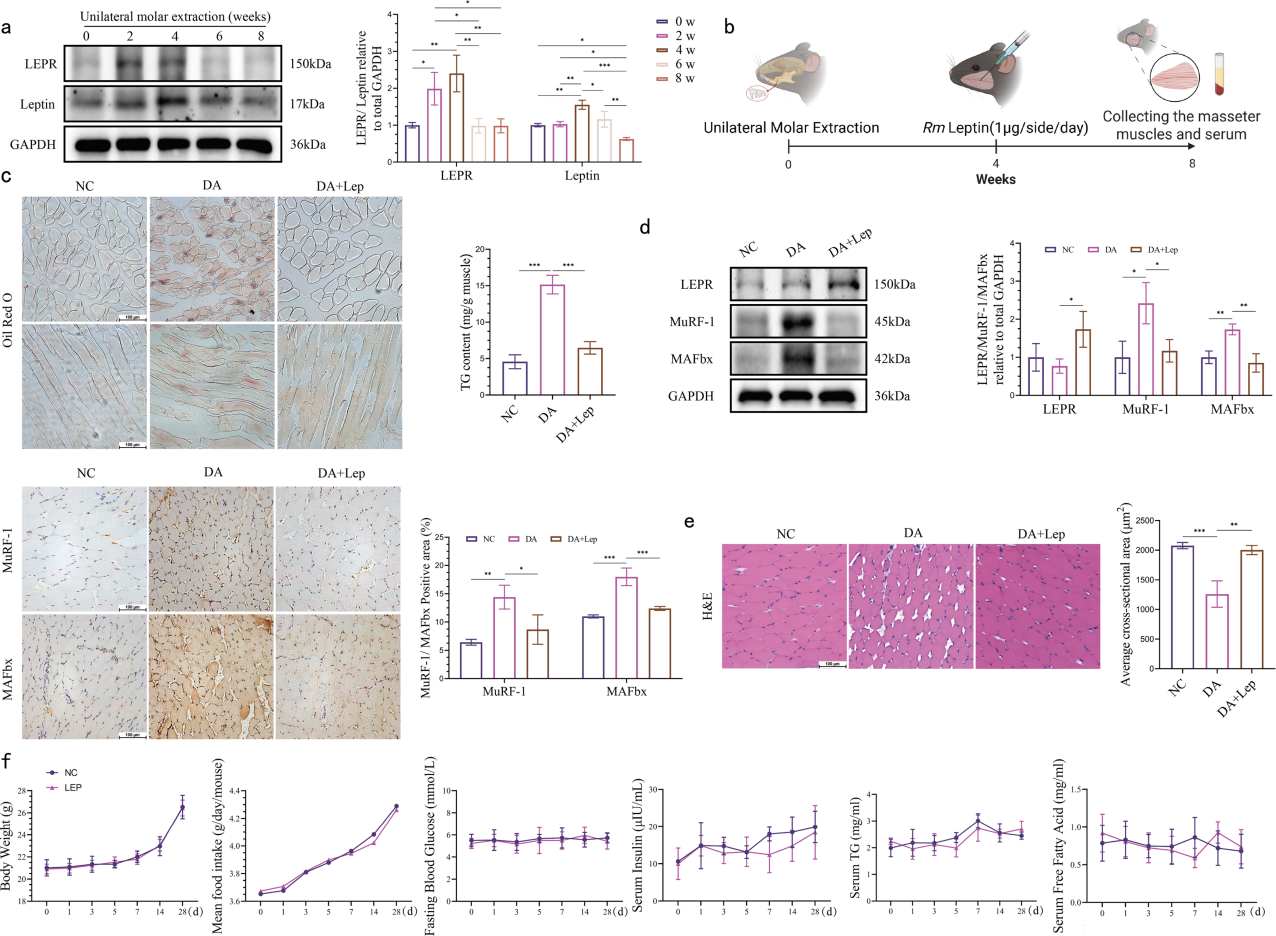
Leptin deficiency as a potential cause of ectopic fat accumulation and muscle atrophy in the masseter muscle. (a) LEPR and leptin bands and quantitative analysis in whole muscle extracts from masseter muscles on the extraction side of mice. GADPH was used as a loading control (one‐way ANOVA; *n* = 3). (b) Experimental flow chart. (c) Representative images of Oil Red O staining of the mouse masseter muscle and triglyceride (TG) content of mouse masseter muscle (scale bar, 100 μm; one‐way ANOVA; *n* = 3). (d) LEPR, MuRF‐1 and MAFbx bands and quantitative analysis in whole muscle extracts of mouse masseter muscle (GADPH was used as a loading control) and images of immunohistochemical staining (MuRF‐1 and MAFbx) of the masseter muscle on the extracted side of mice and quantitative analysis of the positive area (brown colour) (scale bar, 100 μm; one‐way ANOVA; *n* = 3). (e) H&E staining images of masseter muscle on the extraction side of mice and quantitative analysis of the mean cross‐sectional area of muscle fibres (scale bar, 100 μm; one‐way ANOVA; *n* = 3). (f) Mouse body weight, mean food intake, fasting blood glucose, serum insulin, serum TG and serum free fatty acids (one‐way ANOVA; *n* = 3). *p < 0.05, **p < 0.01, ***p < 0.001; error line, mean ± standard value. DA: disuse atrophy Week 8; DA + Lep: DA + exogenous leptin treatment Week 8; NC: negative control.

We next examined the expression changes of leptin and its receptor (LepR) in the masseter muscle throughout the progression of disuse atrophy. Western blot data revealed that the expression of leptin and LepR in the masseter muscle on the operated side was elevated during the early stages of disuse atrophy (less than 4 weeks), potentially as a compensatory response to counteract muscle atrophy and ectopic fat accumulation. However, as the atrophic process progressed, the expression of leptin and LepR gradually decreased and was significantly lower than the initial level at the eighth week, where leptin protein levels in the atrophic masseter muscle were lower than those in the normal masseter muscle (Figure 2a). These findings suggest that the leptin signalling pathway may be inhibited in the later stages of disuse atrophy.

To investigate whether leptin inhibition was the cause of ectopic fat accumulation within the masseter muscle, the injection of exogenous leptin into the masseter muscle at the fourth week of disuse atrophy was performed to sustain elevated leptin levels within the tissue (Figure 2b). After the eighth week of molar extraction, ectopic fat accumulation within the masseter muscle on the operated side was significantly improved in those mice treated with exogenous leptin compared to mice that did not receive leptin. A significant reduction in myocytes that received severe fat infiltration was clearly seen by the Oil Red O staining technique (Figure 2c). Quantification of TG content in the masseter muscle showed a similar trend (Figure 2c), indicating that leptin plays an active role in reducing fat accumulation.

To gain further insight into the specific effects of leptin supplementation on fat metabolism and expression of atrophy‐related proteins within the masseter muscle, immunohistochemical techniques and western blot were utilized to conduct further analyses. The results showed that the expression of MuRF‐1 and MAFbx (Atrogin‐1), two muscle atrophy‐related proteins, was significantly reduced in the masseter muscle of leptin‐treated mice on the operated side compared to controls at the eighth week of disuse atrophy (Figure 2d). This suggests that leptin inhibits the expression of the muscle atrophy‐related factors. In addition, the expression of the leptin receptor (LepR) was observed to be relatively increased following exogenous leptin supplementation (Figure 2d). This result demonstrates that leptin receptors can respond to an increase in leptin to a certain degree, thereby enhancing leptin signalling and promoting the recovery of masseter muscle function. Furthermore, H&E staining of the sections revealed the morphology and arrangement of biting muscle fibres in leptin‐treated mice were significantly restored (Figure 2e). This finding further confirms the potential of leptin in improving the atrophic condition of the masseter muscle and promoting the recovery of muscle tissue. Thus, the extensive ectopic fat deposits observed in the eighth week of disuse atrophic masseter muscle may be directly attributed to leptin deficiency.

In addition, in order to comprehensively assess the systemic metabolic impacts of locally increased leptin levels induced by topical leptin administration, we measured several key parameters in mice, including body weight, food consumption, fasting blood glucose (FBG), serum insulin, serum TG and serum FFA at multiple time points following leptin injection (Figure 2f). The findings revealed that the precisely controlled topical delivery of leptin did not elicit significant changes in these systemic metabolic indicators in the murine model.

### 3.3 RNA‐Seq Reveals Leptin Restores Downregulation of the PPARα/PPAR Signalling Pathway in the Wasted Atrophic Masseter Muscle

**FIGURE 3:**
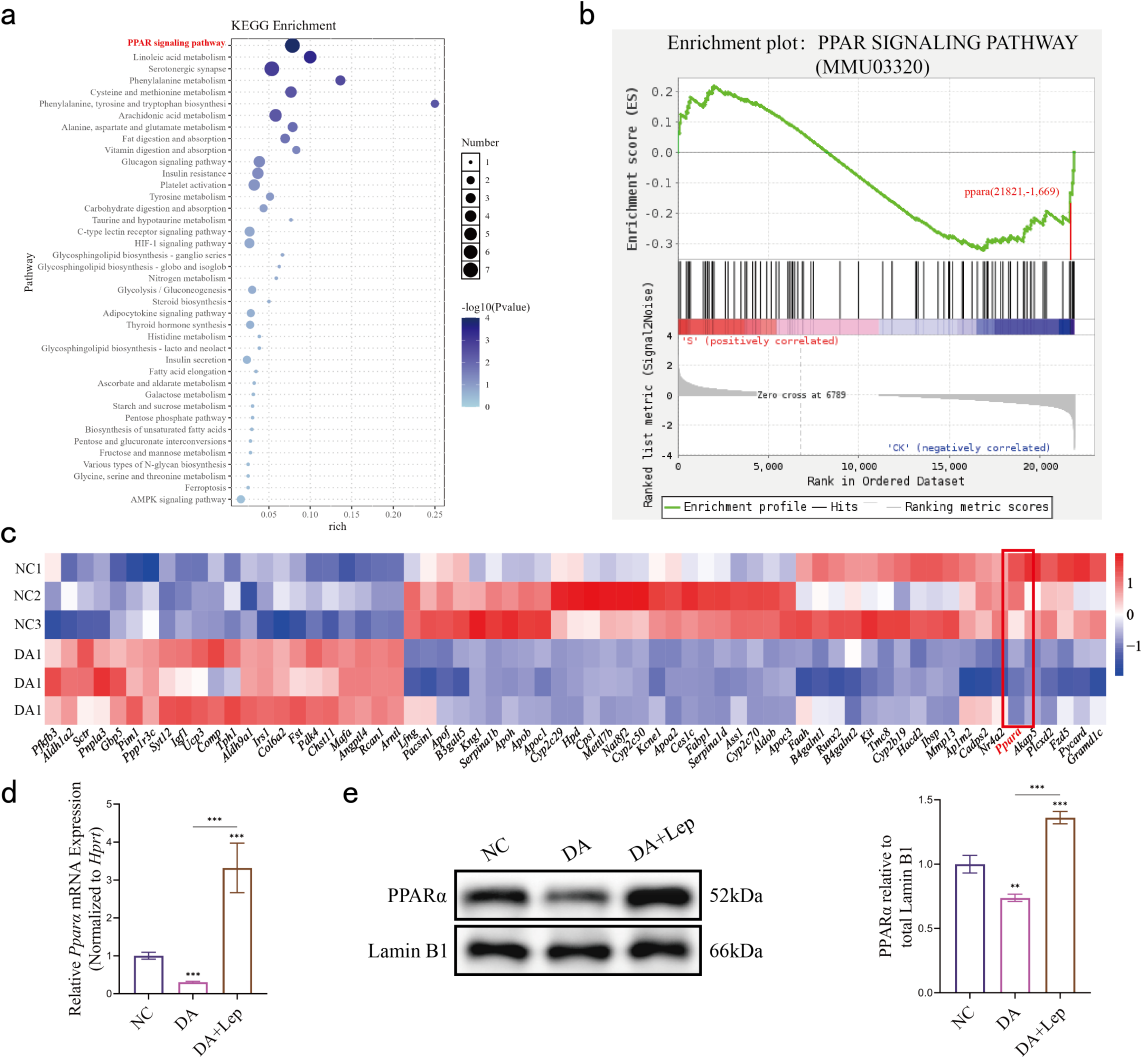
RNA‐seq reveals leptin restores downregulation of the PPARα/PPAR signalling pathway in the wasted atrophic masseter muscle. (a) Bubble plots of KEGG enrichment analysis in normal masseter muscle and atrophied masseter muscle. The significance thresholds of the pathways in the graphs all satisfy *p* < 0.05. (b) GSEA image of the PPARα signalling pathway. (c) Clustering heatmap of differentially expressed genes in lipid metabolism and cytokine‐related pathways in normal masseter muscle and atrophic bite muscle (data are normalized values). (d) qRT‐PCR analysis of PPARα (one‐way ANOVA; *n* = 3). (e) PPARα bands in whole muscle extracts of mouse masseter muscle (Lamin B1 was used as a loading control) and quantitative analysis (one‐way ANOVA; *n* = 3). *p < 0.05, **p < 0.01, ***p < 0.001; error line, mean ± standard deviation. DA: disuse atrophy Week 8; DA + Lep: DA + exogenous leptin treatment Week 8; NC: negative control.

Although the phenotypic effects of leptin in the masseter muscle on ectopic fat accumulation and muscle atrophy have been well characterized, the underlying molecular mechanisms remain unclear. Therefore, we performed transcriptome sequencing of the masseter muscle of mice in the normal and disuse atrophy groups at 8 weeks to identify pathways and genes potentially associated with leptin reduction, leading to fat accumulation and exacerbation of muscle atrophy. KEGG pathway enrichment analysis revealed that a higher number of downregulated genes were associated with cytokine signalling and metabolic pathways. Among these, the PPAR signalling pathway was particularly notable. Additionally, the glycolysis pathway, insulin resistance pathway, AMPK pathway and glucagon signalling pathway, which indirectly regulate fatty acid (FA) metabolism, were also highlighted (Figure 3a). Gene set enrichment analysis (GSEA) revealed that the PPAR signalling pathway was significantly downregulated in the atrophic group compared to the control group. Among them, the PPARA gene, which is closely related to the regulation of lipid metabolism, contributed the main effect of the pathway inhibition. Heatmaps were used to visualize the downregulated genes in these pathways, revealing that the peroxisome proliferator‐activated receptor α (Pparα) gene was significantly downregulated (Figure 3c). The downregulation of these pathways and genes suggests that in the later stages of masseter muscle disuse atrophy, reduced insulin sensitivity, inhibition of glucose uptake and utilization by myocytes and imbalance in PPARα regulation collectively contribute to a reduction in FA oxidation, which in turn leads to the accumulation of TG in myocytes.

To determine the association between the downregulation of PPARα and the decrease in leptin levels, we investigated the mRNA and protein expression levels of PPARα in the masseter muscles of mice treated with leptin. Our findings demonstrated that during the eighth week of masseter muscle atrophy, the PPARα levels in the masseter muscles of leptin‐treated mice were significantly elevated compared to those in the masseter muscles of untreated mice (Figure 3d,e).

### 3.4 Leptin Enhances Mitochondrial FA β‐Oxidation in C2C12 Myotubes by Directly Activating PPARα, Consequently Attenuating Intracellular Lipid Accumulation

**FIGURE 4:**
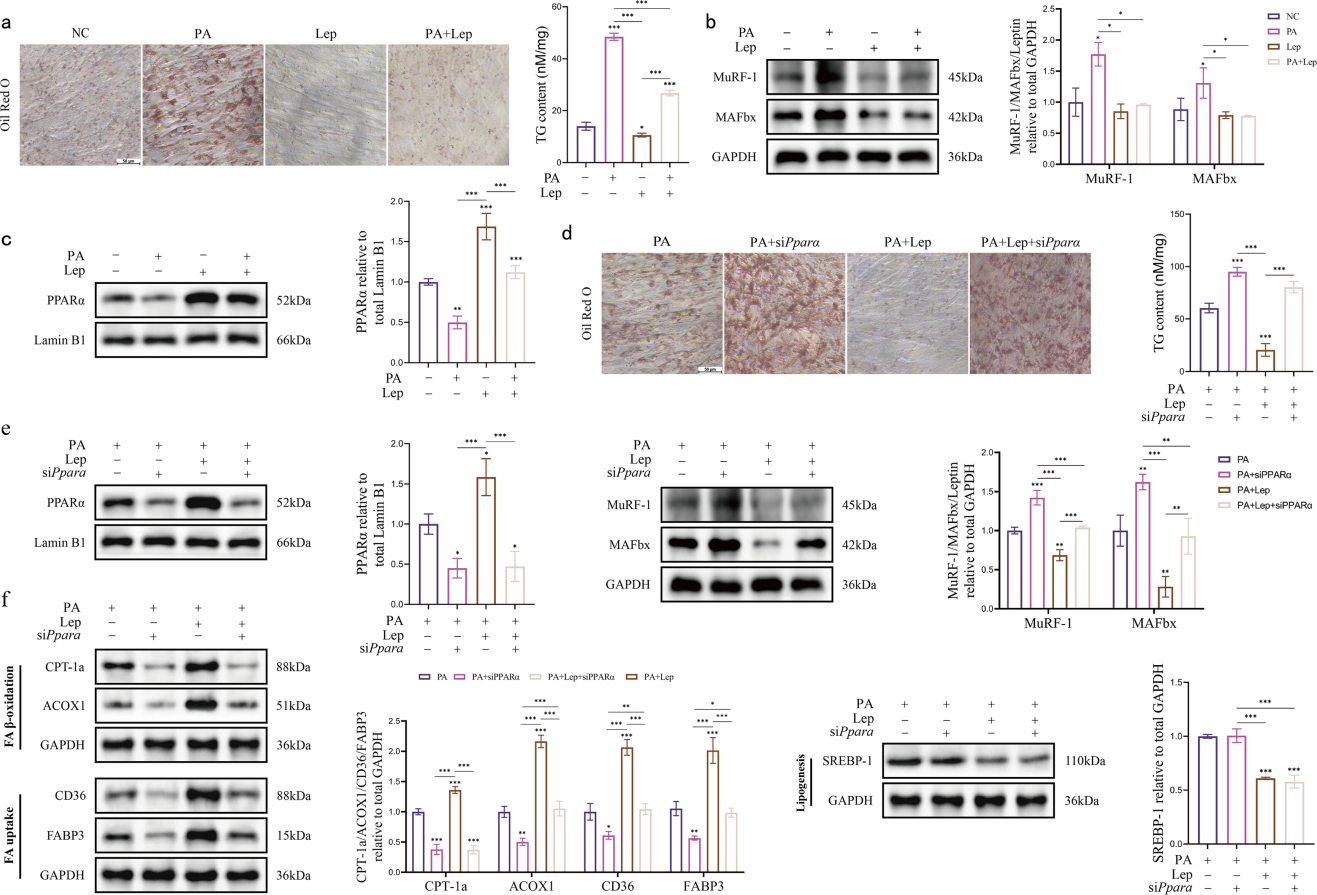
Leptin enhances mitochondrial FA β‐oxidation in C2C12 myotubes by directly activating PPARα, consequently attenuating intracellular lipid accumulation. (a) Oil Red stained images of C2C12 myotubes and triglyceride content (scale bar, 50 μm; one‐way ANOVA; *n* = 3). (b) MuRF‐1 and MAFbx bands and quantitative analysis (one‐way ANOVA; *n* = 3). (c) PPARα bands and quantitative analysis (one‐way ANOVA; *n* = 3). (d) Oil Red stained images of C2C12 myotubes and triglyceride content (scale bar, 50 μm; one‐way ANOVA; *n* = 3). (e) MuRF‐1, MAFbx and PPARα bands and quantitative analysis (one‐way ANOVA; *n* = 3). (f) CPT‐1a, ACOX1, CD36, FABP3 and SREBP‐1 bands and quantitative analysis (one‐way ANOVA; *n* = 3). *p < 0.05, **p < 0.01, ***p < 0.001; error line, mean ± standard deviation. Lep, leptin‐treated group; NC, negative control group (cultured in normal medium); PA, palmitic acid‐treated group; PA + Lep, palmitic acid combined with leptin‐treated group; PA + Lep + siPPARα, palmitic acid, Leptin and PPARα siRNA co‐treatment group; PA + siPPARα: palmitic acid‐treated combined with PPARα siRNA transfection group.

To elucidate the regulatory mechanisms of leptin on lipid metabolism, we initially established a model of palmitic acid (PA)‐induced lipid accumulation in C2C12 myotubes. Oil Red O staining revealed that the lipid droplet area in myotubes was markedly augmented in the PA‐treated group (250 μM, 24 h) when compared to the normal control (NC) group. Conversely, intervention with leptin (50 ng/mL, 24 h) led to a significant reduction in lipid droplet accumulation. This observation was corroborated by the intracellular TG content assay, as depicted in Figure 4a. Simultaneously, western blot analysis demonstrated that upon PA treatment, the expression levels of atrophy‐associated factors MuRF‐1 and MaFbx in myotubes were significantly upregulated. However, leptin effectively mitigated this upregulation, facilitating the recovery of myotubes from atrophy (Figure 4b). Moreover, PA treatment significantly suppressed the protein expression of PPARα, while leptin was able to reverse this inhibitory effect (Figure 4c).

To ascertain whether the function of leptin is contingent upon PPARα, we conducted mechanism validation following the knockdown of PPARα using siRNA. Oil Red O staining results indicated that in the PA + Lep + siPPARα group, the lipid droplet clearance effect of leptin was nearly completely abrogated. Moreover, the intracellular TG content in this group significantly rebounded when compared to the group without PPARα knockdown, as illustrated in Figure 4d. Western blot analysis revealed that treatment with siPPARα led to the abolition of leptin's inhibitory effect on the atrophy‐related proteins MuRF‐1 and MAFbx (Figure 4e), implying that leptin might regulate the muscle atrophy pathway in an indirect manner through PPARα.

Given that PPARα serves as a crucial regulator of lipid metabolism processes, such as FA utilization and oxidation, we explored the impact of leptin on the expression of genes associated with lipid metabolism. Leptin intervention resulted in an upregulation of the expression of proteins involved in FA β‐oxidation and FA uptake, including carnitine palmitoyl transferase 1a (CPT‐1a), acyl‐CoA oxidase 1 (ACOX1), CD36 and FA‐binding protein 3 (FABP3). Concurrently, it led to a downregulation of sterol regulatory element‐binding protein 1 (SREBP‐1), a protein implicated in abortive adipogenesis. Conversely, upon knockdown of PPARα, the knockdown of leptin led to the loss of its stimulatory effect on the proteins involved in FA β‐oxidation and FA uptake, while the inhibitory effect on SREBP‐1 remained intact (Figure 4f).

Thus, leptin could be used to counteracts PA‐induced lipid accumulation in C2C12 myotubes by directly activating the PPARα signalling pathway and upregulating key enzymes involved in FA β‐oxidation and uptake, namely, CPT‐1a, ACOX1, CD36 and FABP3. However, the inhibitory effect of leptin on the lipid‐synthesizing protein SREBP‐1 is independent of PPARα.

### 3.5 FAPs Are a Source of Localized Leptin in the Masseter Muscle

**FIGURE 5:**
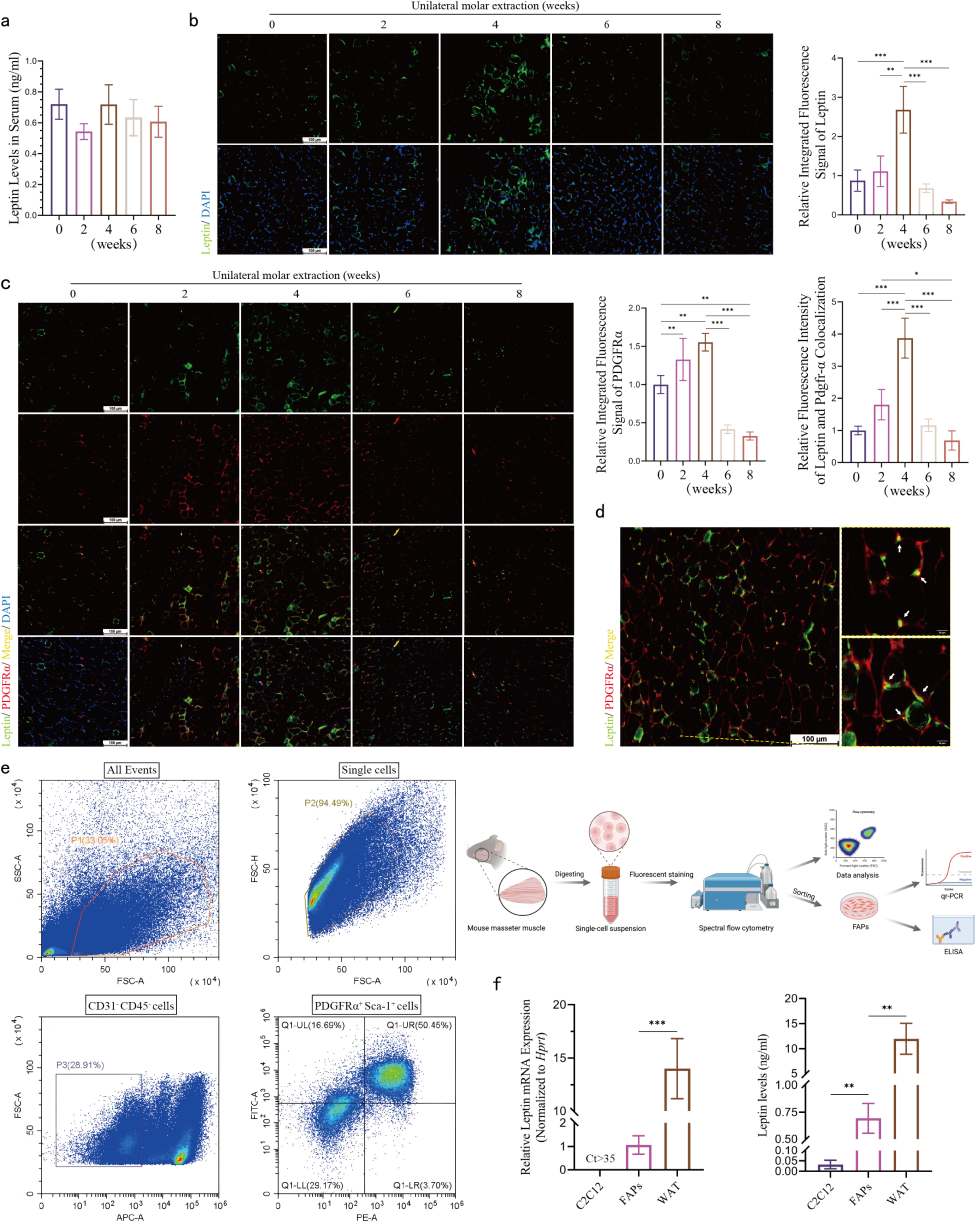
FAPs are a source of localized leptin in the masseter muscle. (a) ELISA assay of serum leptin levels in mice. No significant difference (one‐way ANOVA; *n* = 4). (b) Images of mouse masseter muscle immunofluorescence staining (leptin) and quantitative analysis (scale bar, 100 μm; one‐way ANOVA; *n* = 3). (c) Immunofluorescence staining images and quantitative analysis of leptin and FAPs in mouse masseter muscle (scale bar, 100 μm; one‐way ANOVA; *n* = 3). (d) Histological assessment of mouse masseter muscle sections showing leptin immunoreactivity co‐localized with FAPs. (Right: white arrows). Scale bar = 100 μm (left); scale bar = 10 μm (right). (e) FACS of FAPs. (f) qRT‐PCR and ELISA analysis of leptin (one‐way ANOVA; *n* = 3). *p < 0.05, **p < 0.01, ***p < 0.001; error line, mean ± standard deviation. 0, 2, 4, 6 and 8 weeks: 0, 2, 4, 6 and 8 weeks after unilateral molar extraction.

To investigate the cause of leptin changes during masseter muscle disuse atrophy, the source of leptin in the muscle needs to be clarified. Leptin is a secreted protein, so serum leptin levels in mice were first examined. The ELISA results showed no significant changes in serum leptin levels after surgery (Figure 5a). Thus, masseter muscle disuse atrophy does not affect systemic leptin secretion. Skeletal muscle has been shown to secrete leptin [24]. The increase in leptin in the masseter muscle 4 weeks after the onset of atrophy may be attributed to increased leptin secretion by the muscle. Immunofluorescence staining was used to determine the cellular localization of leptin in skeletal muscle. In mouse masseter muscle, leptin immunoreactivity was weakly detected in the perimyocyte mesenchyme. It was detected in more numerous and intense focal mesenchymal sites during the fourth week of masseter muscle disuse atrophy (Figure 5b). Since FAPs in skeletal muscle have been shown to secrete leptin and FAPs are located as mesenchymal cells in the interstitium of myocytes [25], further co‐localization analyses by immunofluorescence staining showed that immunoreactivity for leptin in mouse masseter muscle was significantly co‐localized with that for PDGFRα^+^ (a marker for FAPs), and both showed a similar trend of increase and decrease in masseter muscle disuse atrophy (Figure 5c,d).

To clarify the function of leptin secretion in FAPs, flow fluorescence sorting technique (FACS) was used to isolate and culture FAPs (Figure 5e). qPCR assay showed that this cell population expressed leptin mRNA, which was about 1/12 of that of white adipose tissue (WAT), whereas no effective expression was detected in the C2C12 myotubes (Ct > 35); ELISA assay confirmed that leptin protein secretion exists in the culture supernatant of FAPs (Figure 5f). The above results confirm that FAPs regulate ectopic lipid accumulation in masseter muscle disuse atrophy by secreting leptin.

### 3.6 Apoptosis of FAPs Affects Leptin Secretion and Exacerbates the Process of Occlusal Disuse Atrophy and Ectopic Fat Accumulation

**FIGURE 6:**
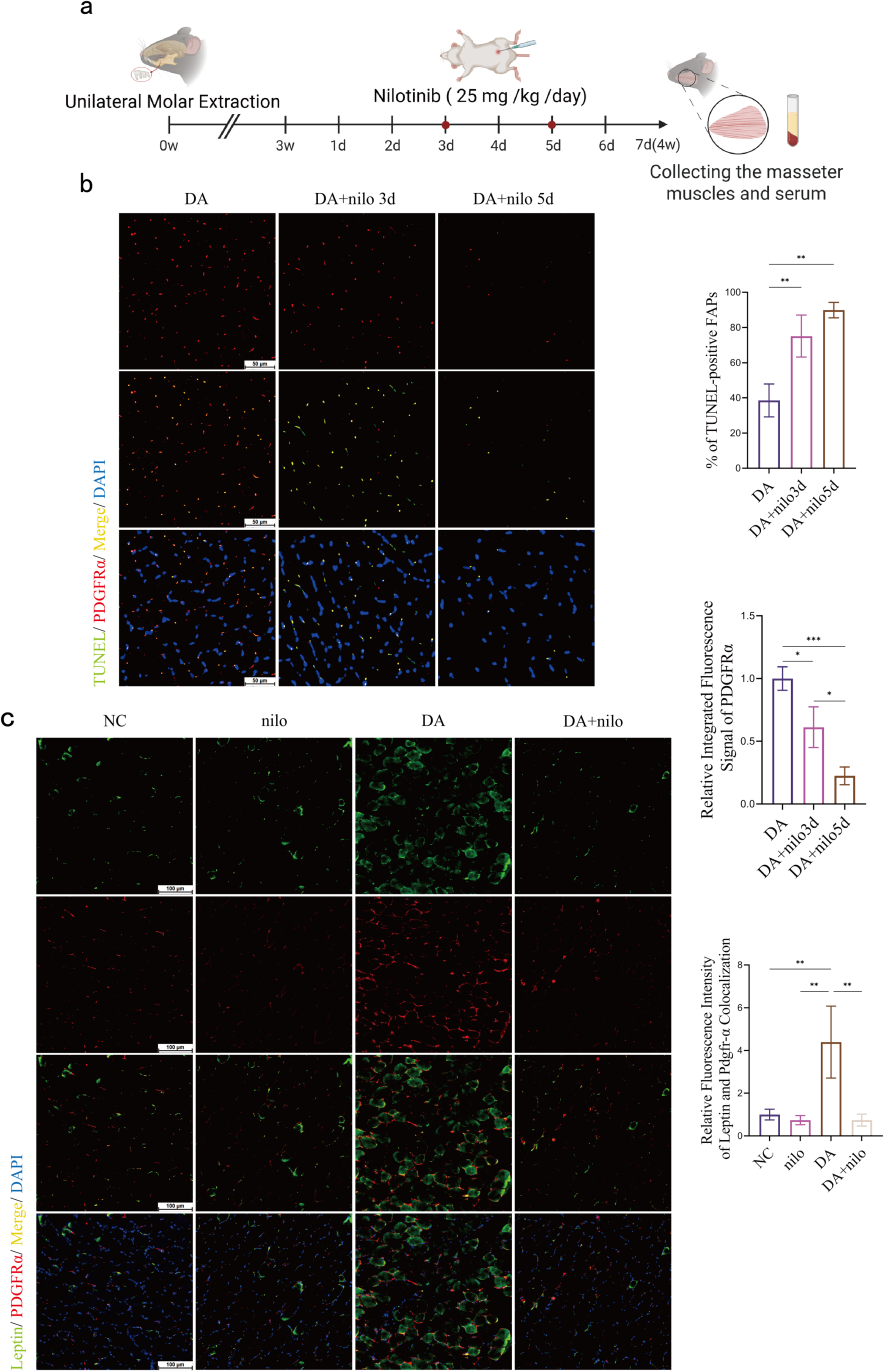
Nilotinib induces apoptosis in masseter muscle FAPs. (a) Experimental flow chart. (b) Effect of continuous injection of nilotinib (25 mg/kg/d ip.) on masseter muscle FAPs apoptosis on Days 3 and 5 in disuse atrophy mice, proportion of apoptotic FAPs cells and quantification of FAPs cells (scale bar, 50 μm; one‐way ANOVA; *n* = 3). (c) Images of masseter muscle immunofluorescence staining and quantitative analysis of leptin and PDGFRα co‐localization (scale bar, 100 μm; one‐way ANOVA; *n* = 3). **p < 0.01, ***p < 0.001. DA: disuse atrophy Week 4; DA + Lep: disuse atrophy Week 4 + 5 days of nilo injection; NC: negative control; nilo: negative control +5 days of nilo injection.

Nilotinib, a tyrosine kinase inhibitor, was originally developed to treat chronic granulocytic leukaemia and functions primarily by inhibiting c‐ABL kinase. Subsequent studies have found that nilotinib also possesses the ability to inhibit the TGF‐β1 signalling pathway [26]. TGF‐β1 is known to promote cell survival [27, 28], whereas nilotinib, by blocking this signalling pathway, significantly accelerates the apoptotic process of FAPs. To determine whether nilotinib affects FAP survival in the masseter muscle, we administered nilotinib intraperitoneally to mice for seven consecutive days until the fourth week after masseter muscle wasting atrophy and assessed apoptosis of FAPs by TUNEL staining. Nilotinib treatment resulted in a significant increase in the frequency of apoptosis of FAPs on Day 3 and a resulting decrease in the total number of FAPs on Day 5 (Figure 6a,b). Thus nilotinib administration influences FAP survival. To investigate leptin secretion after FAP apoptosis, we performed co‐localization analysis using immunofluorescence staining. The results showed that the secretion of leptin in FAP cells and masseter muscle showed a significant decrease with the same trend after induction of apoptosis in FAPs (Figure 6c).

**FIGURE 7:**
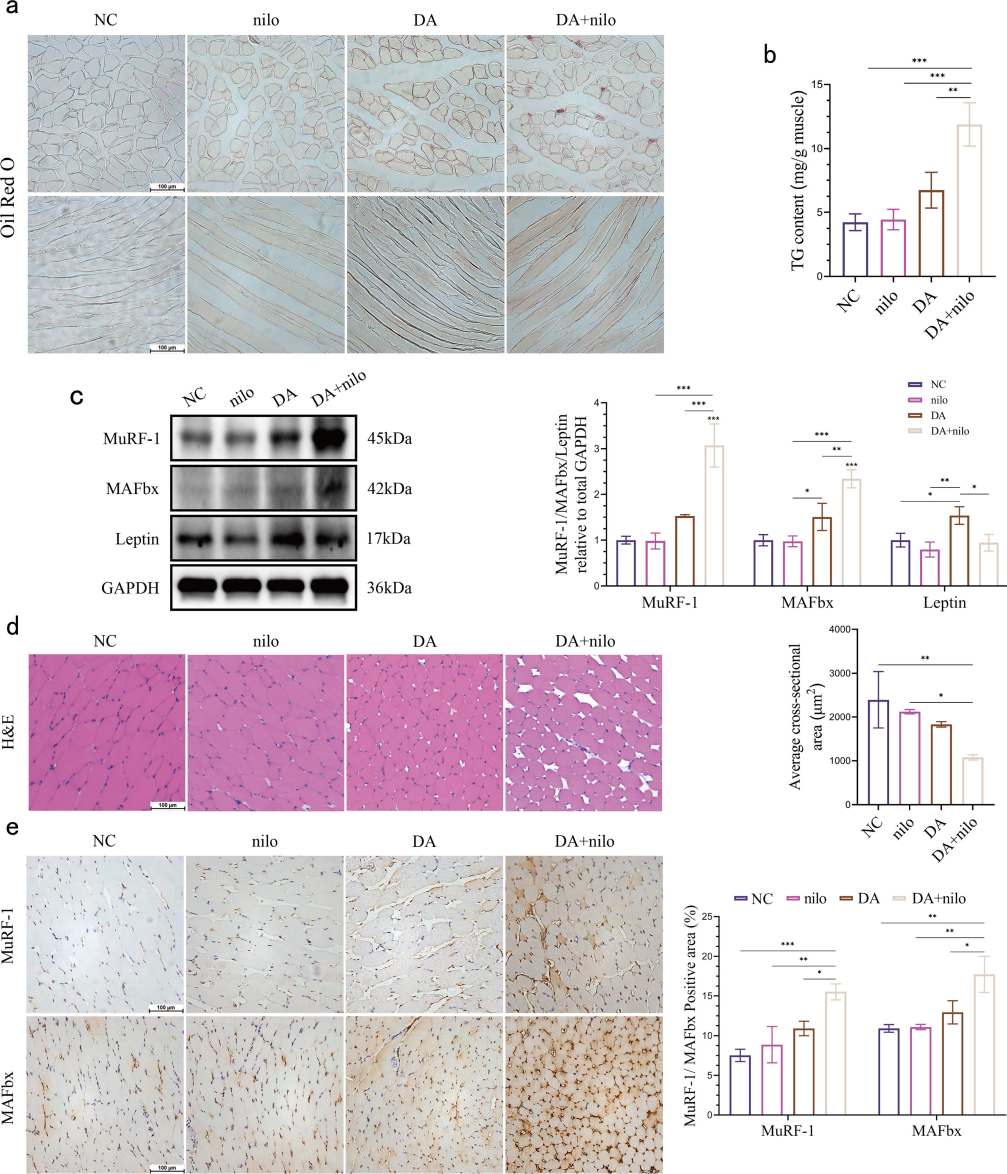
Increased ectopic fat accumulation and wasting atrophy after induction of apoptosis in FAPs cells. (a) Representative images of Oil Red O staining of the mouse masseter muscle (scale bar, 100 μm). (b) Triglyceride (TG) content of mouse masseter muscle (one‐way ANOVA; *n* = 3). (c) Leptin, MuRF‐1 and MAFbx bands in whole muscle extracts of mouse masseter muscle and quantitative analysis. GADPH was used as a control for up‐sampling. (d) H&E staining images of masseter muscle on the extracted side of mice and quantitative analysis of the mean cross‐sectional area of muscle fibres (scale bar, 100 μm; one‐way ANOVA; *n* = 3). (e) Images of immunohistochemical staining (MuRF‐1 and MAFbx) of the masseter muscle on the extracted side of mice and quantitative analysis of the positive area (brown colour) (scale bar, 100 μm; one‐way ANOVA; *n* = 3). *p < 0.05, **p < 0.01, ***p < 0.001; error line, mean ± standard deviation. DA: disuse atrophy Week 4; DA + Lep: disuse atrophy Week 4 + 5‐day nilo injection; NC: negative control; nilo: negative control + 5‐day nilo injection.

Meanwhile, there was an accelerated increase in intramuscular fat accumulation after nilotinib treatment during the fourth week of abortive atrophy by Oil Red O staining and intramasseter muscle TG assays. (Figure 7a,b). The results of western blot data revealed a significant decrease in leptin protein expression in the masseter muscle on the operated side of mice treated with nilotinib, compared to those in the fourth week of abortive atrophy (Figure 7c). H&E staining of the sections revealed that the muscle fibres of the chewing muscle on the surgical side of nilotinib‐treated mice were thinned (Figure 6d), exhibiting an atrophic phenotype. To further understand the specific effects of apoptosis of FAPs on the expression of muscle atrophy‐associated proteins, we examined them using western blot and immunohistochemistry. The results showed that the expression levels of MuRF‐1 and MAFbx, two muscle atrophy‐associated proteins, were significantly elevated (Figure 7c), and the immunohistochemical results showed the same trend (Figure 7e). FAPs in the masseter muscle regulate fat metabolism and muscle atrophy through leptin secretion.

In summary, FAPs regulate fat metabolism in the masseter muscle during disuse atrophy and maintain its function and structure through leptin secretion. In the late stage of disuse atrophy, the lack of FAPs and their leptin secretion may contribute to ectopic fat accumulation and aggravation of atrophy in the masseter muscle.

## 4 Discussion

Disuse atrophy is a significant form of skeletal muscle degeneration, often accompanied by ectopic fat accumulation, a process whose specific mechanisms in the masticatory muscle group remain unclear. In this study, we provide evidence that leptin and its secretory cellular FAPs play a critical role in regulating masseter muscle fat metabolism and muscle atrophy. Notably, the expression of leptin protein showed a trend of increasing and then decreasing during the atrophy of the masticatory muscles. The decrease in leptin was closely linked to increased ectopic fat accumulation and the acceleration of muscle atrophy. Exogenous leptin supplementation reduced ectopic fat accumulation in the disused masseter muscle and facilitated its recovery from atrophy.

Mice were induced to form a masticatory preference by unilateral molar extraction. The same method was used in previous studies, and this model has been validated in rodents and rabbits [29, 30, 31]. Leptin is typically considered a peptide hormone secreted primarily by adipose tissue, acting on the hypothalamus via blood circulation to regulate appetite and metabolic activity [32]. However, the role of leptin extends beyond central regulation. Leptin receptors are widely distributed across various peripheral tissues, including endocrine glands, liver, skeletal muscle, adipose tissue, immune cells and the cardiovascular system. Therefore, the biological role of leptin should not be underestimated [21]. In skeletal muscle, myocytes are the primary target cells of leptin, which directly regulates these cells through leptin receptors [33]. It should be noted that skeletal muscle uptake of circulating leptin is relatively low [34], which implies that local leptin production may exert a significant influence on skeletal myocyte lipid metabolism that has previously been attributed to circulating leptin. It is noteworthy that although there was an increase in leptin receptors following leptin supplementation, the extent of this increase was limited. This is due to prolonged exposure to excessive leptin, which may suppress leptin receptor expression or reduce its signalling capacity, leading to reduced sensitivity of target cells to leptin signalling. Therefore, the therapeutic role of leptin in treating disuse atrophy is constrained in the context of our current study.

Leptin is not only secreted by adipose tissue but also by skeletal muscle. Previous studies have shown that leptin mRNA is expressed in both mouse and human limb skeletal muscle [24, 25, 35]. Moreover, Emmanuel Nwadozi et al. demonstrated that leptin production in skeletal muscle originates not from satellite cells or skeletal myocytes, but from PDGFRα^+^ perivascular cells with lipogenic potential, referred to as FAPs with PDGFRα^+^ as a cellular marker. In our study, fluorescence co‐localization analysis of FAPs combined with FACS confirmed their leptin‐secreting ability in an experimental model.

FAPs maintain a certain number and activity in a normal environment, providing critical functional support to satellite cells and contributing to the maintenance of muscle tissue homeostasis [36]. However, under different pathological conditions, dysregulation of FAP activity occurs differently: In the muscles of patients with Duchenne muscular dystrophy (DMD) and long‐term radiation exposure, FAPs over‐differentiate into fibroblasts, causing fibrosis in skeletal muscle [37, 38]. On the other hand, FAPs can rapidly increase in the short term and differentiate into adipocytes under the regulation of Pparγ in a mouse model of muscle injury induced by glycerol injection [39, S1]. Notably, in the cardiotoxin injection‐induced muscle regeneration model, the number of PDGFRα^+^ cells increased significantly initially but decreased and did not differentiate into adipocytes as muscle regeneration proceeded. This is due to the inhibitory effect of myofibers on the adipogenic differentiation of PDGFRα^+^ cells [S2]. Thus it can be deduced that the direction of FAP differentiation depends primarily on the surrounding microenvironment.

In this study, we systematically revealed the dynamic regulatory role of fibro‐adipogenic progenitor cells (FAPs) and their secreted factor leptin in the imbalance of muscle fat metabolism by constructing a mouse masseter muscle disuse atrophy model. We found that during the early stages of atrophy (0–4 weeks), inflammatory responses in the local microenvironment may trigger the activation and expansion of FAPs, a process similar to the reparative response after acute muscle injury [S3–5]. Activated FAPs exert compensatory protective effects by secreting leptin, and the mechanism may involve the inhibitory effect of leptin on the ubiquitin‐proteasome system. However, unlike the acute injury model, since disuse atrophy did not result in extensive destruction of muscle fibres, and transcriptome analysis showed that the expression of Pparγ, a key regulator of adipose differentiation, was not significantly upregulated (Figure 4F), it is suggested that the microenvironment lacks the necessary signals (e.g., mechanical stresses or adipogenic cytokines) that drive the differentiation of FAPs to mature adipocytes [S3, 6]. This persistent functional inhibition eventually leads to a progressive loss of functional activity and a decrease in the number of FAPs, which in turn triggers a decrease in local leptin levels. The reduction in leptin secretion may exacerbate the abnormal accumulation of TG in myocytes by inhibiting PPARα‐mediated FA β‐oxidation and FA uptake.

Although experiments demonstrating nilotinib‐induced apoptosis in FAPs have confirmed that FAPs are the primary source of localized leptin in the masseter muscle, the issue of its cell specificity remains open for discussion. While this study confirms nilotinib exhibits strong selectivity for FAPs with minimal off‐target effects (Figure S3), our exploration of nilotinib remains incomplete. Nilotinib serves as a valuable tool in preliminary studies; future work utilizing genetic models is essential to validate our findings. Furthermore, the regulatory mechanisms underlying the reduction of FAPs in late‐stage skeletal muscle atrophy remain to be elucidated. Existing research suggests that FAP homeostasis imbalance involves multiple pathways, such as Hippo pathway inhibition impairing their activation and regenerative potential [S7], and elevated ROS levels triggering mitochondrial dysfunction and senescent apoptosis [S8]. However, this study did not delve into the specific mechanisms underlying the reduction in FAPs within the disused atrophied masticatory muscles.

Notably, this study focused on elucidating the direct role of leptin in FA catabolism within the masticatory muscles. The observed reduction in leptin receptor responsiveness—potentially due to receptor desensitization from prolonged exposure to high leptin concentrations—warrants further investigation. Future studies may explore strategies such as intermittent dosing or combining leptin with sensitizers like mitochondrial function activators or AMPK agonists. These approaches could help overcome desensitization effects and enhance therapeutic efficacy. Although this study utilized a mouse model, the concurrent expression of FA synthase and leptin in human skeletal muscle supports its relevance to human physiology. Similar patterns of fat infiltration and muscle atrophy have been observed in patients with disuse muscle atrophy caused by prolonged bed rest or joint immobilization. Thus, the core role of the FAPs‐leptin‐PPARα axis in regulating intramuscular lipid metabolism, as revealed in this study, provides important insights into the pathophysiological mechanisms underlying human disuse muscle atrophy. It also lays the foundation for translating these findings into novel therapeutic strategies.

In conclusion, this study provides an in‐depth exploration of masseter muscle disuse atrophy in mice, establishing that ectopic fat formation in the masseter muscle is linked to insufficient local leptin expression. It also demonstrates that leptin‐secreting cells, FAPs, play a role in regulating fat metabolism in the masseter muscle. In addition, FAPs were stimulated to be recruited in the pre‐disuse atrophy phase and decreased in the late phase suggesting the involvement of pericytes in adaptive changes in the masseter muscle. Finally, considering the diverse roles of leptin in peripheral tissues, local leptin production may serve as an intrinsic mechanism to promote energy homeostasis and cellular communication within masseter muscles by regulating fat metabolism in myocytes. In conclusion, our findings suggest that disuse atrophy involves not only diminished muscle activity but also disturbances in fat metabolism. Addressing this imbalance could aid in the prevention and treatment of disuse atrophy and provide insights into other skeletal muscle atrophy disorders.

## Funding

This study was supported by the Anhui Province Scientific Research Preparation Program Project (2022AH050734) and the ‘Feng Yuan’ Program (2022xkfyhz03 and 2023xkfytszd02). We would like to acknowledge BioRender (https://www.biorender.com/) for providing the tools and resources used to generate the schematic diagrams presented in this study (e.g., Figures 1a, 2b, 5e and 6a).

## Ethics Statement

The study was conducted in strict accordance with international ethical guidelines and the National Institutes of Health Guide for the Care and Use of Laboratory Animals, and all experimental treatments were approved by the Institutional Animal Care and Use Committee of Anhui Medical University [Ethical Approval Number: LLSC20231010].

All experimental procedures were conducted at Anhui Medical University, Hefei, China, under the supervision of qualified personnel. Animals were housed and cared for in accordance with the principles of animal welfare to ensure that they received appropriate food, water, shelter and environmental enrichment to promote overall animal health. The experiments were designed to minimize animal discomfort, suffering and the number of animals used while achieving the scientific objectives of the study. Animals were analgesized and anaesthetized as necessary throughout the experiment, and every effort was made to ensure that the animals were treated humanely at every stage.

The authors declare that no animals were unnecessarily harmed during the course of this study, and that all experimental procedures complied with the ethical standards set by the Animal Protection and Utilization Committee of Anhui Medical University and relevant Chinese regulations. The findings reported in this manuscript are based on data collected in a scientifically rigorous and ethically responsible manner.

The manuscript does not contain clinical studies or patient data.

## Conflicts of Interest

The authors declare no conflicts of interest.

## Supporting information


**Figure S1:** (a) Serum leptin levels at 0.5, 1, 2, 6, 12 and 24 h after leptin injection (*n* = 3). 0.01,0.1,1: The concentrations of injected leptin solution were 0.01, 0.1 and 1 μg/μL, respectively. (b) Oil red O staining and HE staining of masseter muscle sections at different concentrations of leptin injected for 4 weeks after leptin injection. (c) UMAP images and quantified percentages representing different cell types in rat masseter muscle. (d) AMPK and p‐AMPK bands in whole muscle extracts from mouse masseter muscles (GAPDH was used as up‐sampling control) and quantitative analysis (one‐way ANOVA; *n* = 3). *p < 0.05, **p < 0.01, ***p < 0.001; error lines, mean ± standard deviation. NC: negative control; DA: disuse atrophy week 8; DA + Lep: DA + exogenous leptin treatment Week 8.

**Figure S2:** (a) Mean food intake, fasting blood glucose, serum insulin, serum TG and serum free fatty acids (one‐way ANOVA; *n* = 3). 0, 2, 4, 6 and 8 weeks: 0, 2, 4, 6 and 8 weeks after unilateral molar extraction. (b) Leptin levels in the masseter muscle at 0.5, 1, 2, 6, 12 and 24 h after leptin injection(*n* = 3). 0.01,0.1,1: The concentrations of injected leptin solution were 0.01, 0.1 and 1 μg/μL, respectively.

**Figure S3:** Apoptosis of FAPs cells (PDGFRα), muscle fibres (dystrophin), satellite cells (Pax7), endothelial cells (CD31) and macrophages (F4/80) in the masseter muscle of mice with disuse atrophy following continuous administration of nilotinib (25 mg/kg/d, intraperitoneal injection) on Day 3. (Representative image: scale bar, 10 μm).

**Data S1:** Supporting information.
