# Supplementary figures and images for: Leptin From Fibro‐Adipogenic Progenitor Cells (FAPs) Regulates Masseter Muscle Disuse Atrophy and Ectopic Fat Accumulation
Source: J Cachexia Sarcopenia Muscle. 2025 Nov 26;16(6):e70141. doi: 10.1002/jcsm.70141 (PMC12657642; doi:10.1002/jcsm.70141)

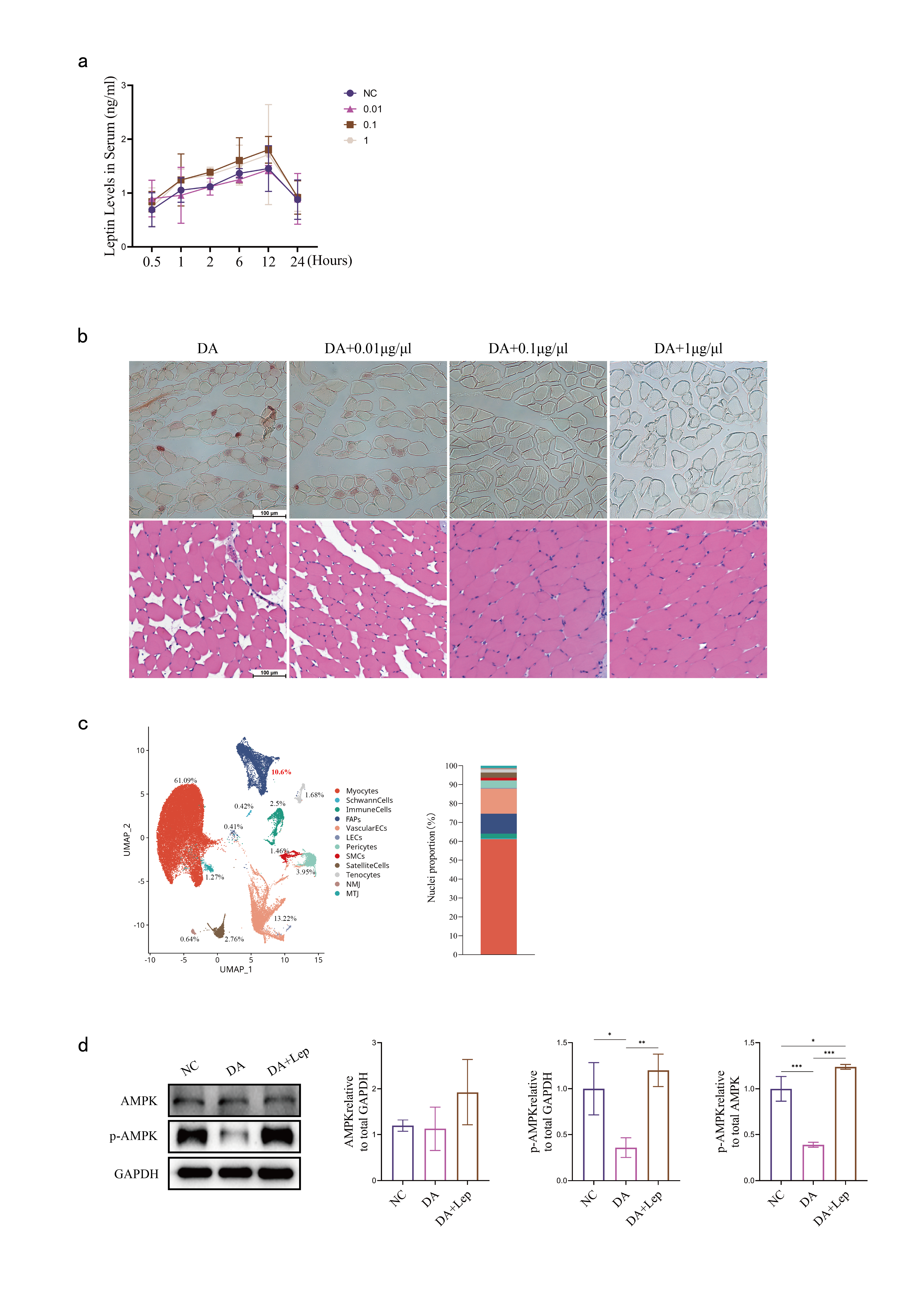

Supplement: Supplementary file 1 — Figure S1: (a) Serum leptin levels at 0.5, 1, 2, 6, 12 and 24 h after leptin injection (n = 3). 0.01,0.1,1: The concentrations of injected leptin solution were 0.01, 0.1 and 1 μg/μL, respectively. (b) Oil red O staining and HE staining of masseter muscle sections at different concentrations of leptin injected for 4 weeks after leptin injection. (c) UMAP images and quantified percentages representing different cell types in rat masseter muscle. (d) AMPK and p‐AMPK bands in whole muscle extracts from mouse masseter muscles (GAPDH was used as up‐sampling control) and quantitative analysis (one‐way ANOVA; n = 3). *p < 0.05, **p < 0.01, ***p < 0.001; error lines, mean ± standard deviation. NC: negative control; DA: disuse atrophy week 8; DA + Lep: DA + exogenous leptin treatment Week 8. [file JCSM-16-e70141-s004.png]

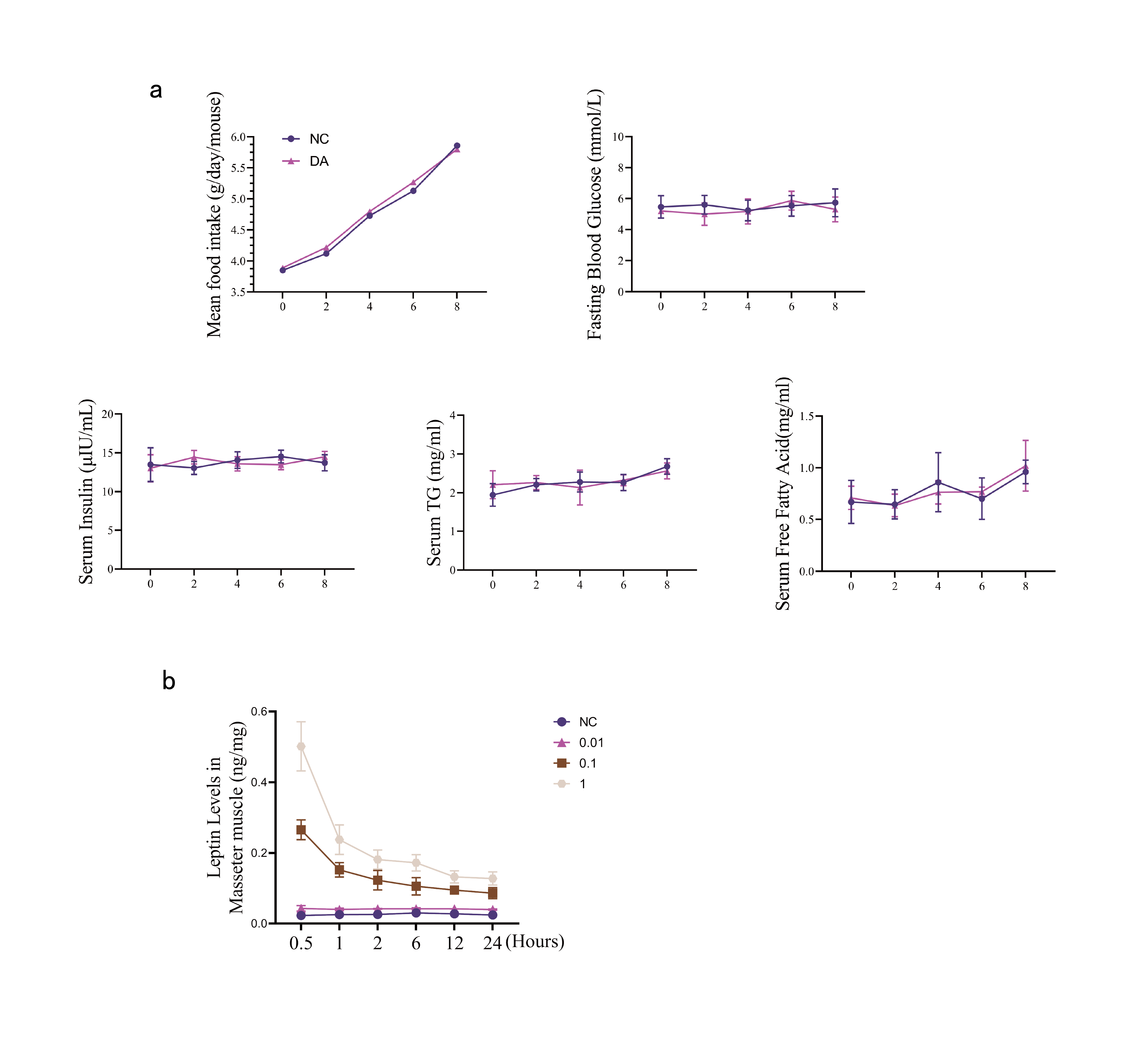

Supplement: Supplementary file 2 — Figure S2: (a) Mean food intake, fasting blood glucose, serum insulin, serum TG and serum free fatty acids (one‐way ANOVA; n = 3). 0, 2, 4, 6 and 8 weeks: 0, 2, 4, 6 and 8 weeks after unilateral molar extraction. (b) Leptin levels in the masseter muscle at 0.5, 1, 2, 6, 12 and 24 h after leptin injection(n = 3). 0.01,0.1,1: The concentrations of injected leptin solution were 0.01, 0.1 and 1 μg/μL, respectively. [file JCSM-16-e70141-s001.png]

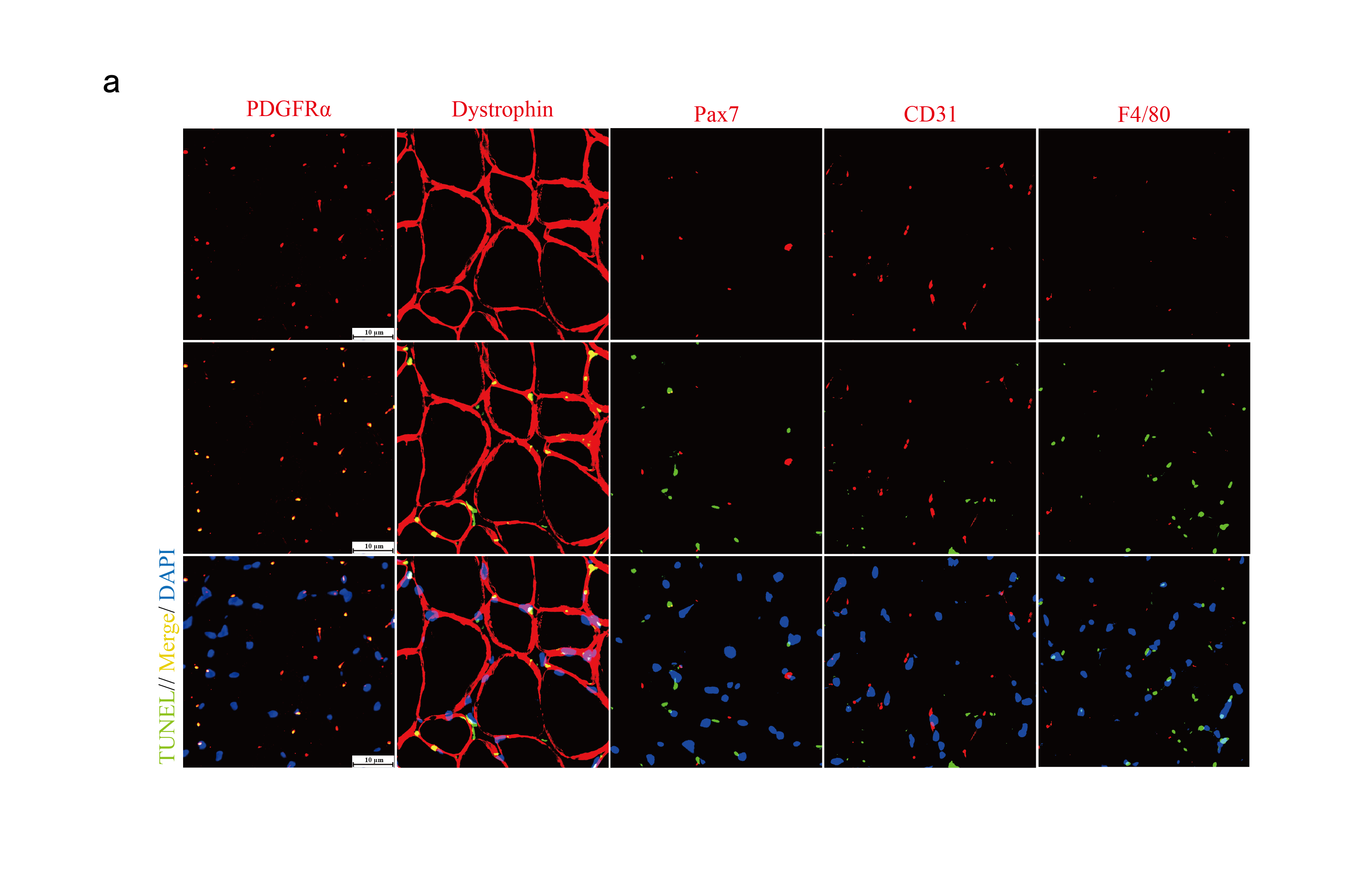

Supplement: Supplementary file 3 — Figure S3: Apoptosis of FAPs cells (PDGFRα), muscle fibres (dystrophin), satellite cells (Pax7), endothelial cells (CD31) and macrophages (F4/80) in the masseter muscle of mice with disuse atrophy following continuous administration of nilotinib (25 mg/kg/d, intraperitoneal injection) on Day 3. (Representative image: scale bar, 10 μm). [file JCSM-16-e70141-s003.png]
