# Supplementary material for: Leptin From Fibro‐Adipogenic Progenitor Cells (FAPs) Regulates Masseter Muscle Disuse Atrophy and Ectopic Fat Accumulation
Source: J Cachexia Sarcopenia Muscle. 2025 Nov 26;16(6):e70141. doi: 10.1002/jcsm.70141 (PMC12657642; doi:10.1002/jcsm.70141)
Supplement: Supplementary file 4 — Data S1: Supporting information. [file JCSM-16-e70141-s002.docx]

**Additional references**

1. **Reggio A, Rosina M, Palma A, et al. Adipogenesis of skeletal muscle fibro/adipogenic progenitors is affected by the WNT5a/GSK3/β-catenin axis. *Cell Death Differ*. 2020;27(10):2921-2941. doi:[10.1038/s41418-020-0551-y](https://doi.org/10.1038/s41418-020-0551-y)**
2. **Uezumi A, Fukada S ichiro, Yamamoto N, Takeda S, Tsuchida K. Mesenchymal progenitors distinct from satellite cells contribute to ectopic fat cell formation in skeletal muscle. *Nat Cell Biol*. 2010;12(2):143-152. doi:[10.1038/ncb2014](https://doi.org/10.1038/ncb2014)**
3. Giuliani G, Rosina M, Reggio A. Signaling pathways regulating the fate of fibro/adipogenic progenitors (FAPs) in skeletal muscle regeneration and disease. *FEBS J*. 2022;289(21):6484-6517. doi:[10.1111/febs.16080](https://doi.org/10.1111/febs.16080)
4. Joe AWB, Yi L, Natarajan A, et al. Muscle injury activates resident fibro/adipogenic progenitors that facilitate myogenesis. *Nat Cell Biol*. 2010;12(2):153-163. doi:[10.1038/ncb2015](https://doi.org/10.1038/ncb2015)
5. Wosczyna MN, Konishi CT, Perez Carbajal EE, et al. Mesenchymal Stromal Cells Are Required for Regeneration and Homeostatic Maintenance of Skeletal Muscle. *Cell Rep*. 2019;27(7):2029-2035.e5. doi:[10.1016/j.celrep.2019.04.074](https://doi.org/10.1016/j.celrep.2019.04.074)
6. Chen W, You W, Valencak TG, et al. Bidirectional roles of skeletal muscle fibro-adipogenic progenitors in homeostasis and disease. Ageing Res Rev. 2022;80:101682. doi:10.1016/j.arr.2022.101682
7. Cruz-Soca M, Faundez-Contreras J, Córdova-Casanova A, et al. Activation of skeletal muscle FAPs by LPA requires the Hippo signaling via the FAK pathway. Matrix Biol. 2023;119:57-81. doi:10.1016/j.matbio.2023.03.010
8. Yao Y, Luo Y, Liang X, et al. The role of oxidative stress-mediated fibro-adipogenic progenitor senescence in skeletal muscle regeneration and repair. Stem Cell Res Ther. 2025;16(1):104. Published 2025 Mar 1. doi:10.1186/s13287-025-04242-4
